# Analysis of the Selection Signal of the Tibetan Black Chicken Genome Based on Whole-Genome Sequencing

**DOI:** 10.3390/genes14091672

**Published:** 2023-08-24

**Authors:** Jing Feng, Wei Zhu, Hairen Shi, Da Peng, Lei Zang, Yan Wang, Luobu ZhaXi, Jiancai BaiMa, Felix Kwame Amevor, Xiaoqi Wang, Xueying Ma, Xiaoling Zhao

**Affiliations:** 1 Institute of Animal Husbandry and Veterinary Medicine, Tibet Academy of Agricultural and Animal Husbandry Science, Lhasa 850009, China; shihr21@lzu.edu.cn (H.S.); 13658917784@163.com (D.P.); wangyanls@hotmail.com (Y.W.); maxueying006@126.com (X.M.); 2 Key Laboratory of Animal Genetics and Breeding on Tibetan Plateau, Ministry of Agriculture and Rural Affairs, Lhasa 850009, China; 3 State Key Laboratory of Swine and Poultry Breeding Industry, College of Animal Science and Technology, Sichuan Agricultural University, Chengdu 611130, China; weizhu451@163.com (W.Z.); amevorfelix@gmail.com (F.K.A.); 4 Farm Animal Genetic Resources Exploration and Innovation Key Laboratory of Sichuan Province, Sichuan Agricultural University, Chengdu 611130, China; 5 College of Pastoral Agriculture Science and Technology, Lanzhou University, Lanzhou 730020, China; 6 Shannan Longzi County Agriculture and Animal Husbandry Comprehensive Service Center, Shannan 856600, China18791322009@163.com (J.B.); 7 Agriculture and Animal Husbandry Comprehensive Service Center of Lazi County, Shigatse 858100, China; 13908921647@163.com

**Keywords:** Tibetan black chicken, whole-genome sequencing, selection signal, calcium signaling pathway, circadian entrainment functional analysis

## Abstract

Background: The Tibetan chicken has adapted well to high altitudes genetically after its long-term habitation in the plateau. In this study, we analyzed the selection signal of Tibetan black chickens (TBCs) and discovered genes associated with the characteristics of germplasm. Methods: Whole-genome sequencing (WGS) was used to identify the single-nucleotide polymorphism (SNP) markers and genetic structures in the genome of Tibetan black chickens. Further, we performed a comparative population genomics analysis between the genomic data obtained in this present study and the genomic data for five wild red jungle fowls (RJFs) accessed from the NCBI database (GenBank accession number PRJNA241474). Thereafter, the Fst and *Pi* selections were used to identify genes under positive selection in the Tibetan black chicken genome. Results: A total of 9,490,690 SNPs were identified in the Tibetan black chickens. In addition, the results from the gene ontology (GO) analysis showed that 732 genes of TBCs were enriched in a total of 210 GO terms with specific molecular functions such as regulation of cellular catabolic process, the MAPK signaling pathway, regulation of ion transport, growth, morphogenesis and lung alveolus development which may provide a better mechanism to facilitate oxygen transport and utilization in TBCs. Moreover, the results from the KEGG analysis showed that 732 genes of the TBCs were significantly enriched in the calcium signaling pathway, circadian entrainment (*ADCY1*, *GNG7* and *PER3*), oxytocin signaling pathway and pathways of multiple neurodegeneration diseases. In addition, the CD86 antigen (*CD86*) was identified as a gene associated with the immune response in chickens. It was also revealed that genes such as *TRIT1*, *HPCAL4*, *NT5C1A* and *HEYL* were discovered under selection in Tibetan black chickens on chromosome 23. These genes may be related to the local adaptive characteristics of Tibetan black chickens, for instance, *NT5C1A* and *HEYL* may be involved in the high-altitude adaption of oxygen delivery in Tibetan black chickens. Conclusions: In summary, we found that selection mainly affects the disease resistance and cold acclimatization of Tibetan black chickens. Hence, these results may provide important genetic information for the evolution and breeding of Tibetan black chickens.

## 1. Introduction

The Tibetan Plateau is one of the harshest environments for human and animal settlements, with an elevation ranging from 2200 to 4100 m. Both humans and animals in Tibetan settlements have survived harsh conditions such as low oxygen levels by undergoing specific physiological and genetic adaptations [1,2].

The Tibetan chicken is one of the chicken breeds found in the plateau region, and it is unique to the Qinghai–Tibet Plateau in China [3]. The Tibetan black chicken (TBC) is a small-sized primitive local chicken species which is distributed in the semi-agricultural and semi-pastoral areas of the Qinghai–Tibet Plateau of Lhunzi County, Shannan Region, China [1,2]. Tibet has an altitude of about 3900 m (28°07′ N–28°52′ N, 91°53′ E~93°06′ E), an annual average temperature of about 5.5 °C and average annual sunshine hours of 3005 h. Tibetan black chickens can survive and develop very well in an oxygen-deficient, severe, cold environment. This shows that Tibetan black chickens are resistant to harsh weather conditions and food scarcity [4]. Reports have indicated that the genotype of this chicken breed enables them to adapt to environments with low oxygen and low pressure [1,4]. The Tibetan black chicken is a suitable breed for the modern poultry industry due to its excellent qualities, such as resistance to diseases and harsh weather conditions and their adaptability to low-quality feeds, as well as their production of high-quality meat and eggs [5]. The Tibetan black chicken is a desirable local chicken breed in China; however, reports on the factors associated with their high quality and productivity are limited. Screening for unique functional genes of chickens by high-throughput sequencing can help to select specific breeds for desirable traits, such as high egg production rates and good meat quality, thereby promoting their economic value using modern breeding techniques [6].

Selective sweep explains how alleles for favorable adaptations and their associated chromosomal alleles become more frequently observed in a population due to natural selection [7]. This is initiated by the mutational origin of an advantageous allele [7]. Therefore, selective sweep at some genetic sites reduces the degree of polymorphism when certain genes are subjected to excessive positive selection [5,6,8,9,10,11]. During the occurrence of beneficial mutation, the mutated site is selected, making the frequency of such a mutation inevitably increase in the population. When such positive selection occurs, other polymorphic sites with specific haplotypes close to the mutation are also selected. Therefore, the overall genetic variation of the region surrounding the mutation is reduced due to an increase in the proportion of a specific haplotype in the population [12].

In this study, the fixation index (FST) was employed in whole-genome resequencing in order to identify and select signatures for selection in TBC. The fixation index (FST) is an outlier method used to identify genomic regions involved in speciation [13]. Whole-genome resequencing is a powerful tool for genetic assessment, selective scan analysis and genetic relationship exploration. Previous studies have reported the genetic characteristics of high-altitude adaptation in Tibetan chickens by comparing the genomic regions of strongly selected traits between the Tibetan chickens and low-altitude local chickens [14,15,16]. This study was conducted to determine whether selection played a key role in this specific region by comparing different sites at the genomic level. The Tibetan chicken is a kind of semidomesticated bird living in the Qinghai–Tibet Plateau in the west of China, mainly distributed over a large area with altitudes from 2200 to 4100 m. Its appearance and behavior are very similar to the red jungle fowl [17]. The Tibetan chicken has adapted well to the high altitude genetically after its long-term habitation in the plateau [17].

To identify the selection signatures of TBCs resulting from their domestication, we performed whole-genome resequencing on 30 TBCs. Furthermore, using the data obtained, together with five publicly available genomes of RJF accessed from the National Center for Biotechnology Information (NCBI), a comprehensive analysis of genetic variants was performed to identify the genomic regions under selection in TBC. This present study provides genetic information which is beneficial for breeding, and also reveals the evolutionary history of the TBC breed. In addition, identifying the genetic loci related to economic traits provides a rational basis for further studies on chicken breeding.

## 2. Materials and Methods

### 2.1. Sampling and Whole-Genome Resequencing

In this study, a total of 30 healthy female Tibetan black chickens (TBCs) at a similar age (20 weeks) reared in the same environment were used. Blood samples (2 mL) were collected (via the wing vein) from the 30 female Tibetan black chickens (Lhunzi County, Tibetan). Subsequently, total genomic DNA was isolated from the blood using Qiagen DNeasy Blood and Tissue Kits following the prescribed protocols. Then, the DNA concentration was finalized to 200 ng/µL using sterile water. The 150-bp paired-end (PE) libraries were prepared for sequencing on an Illumina HiSeq X Ten platform.

We further conducted a comparative population genomics analysis using our obtained data and genomic data from five wild red jungle fowls (RJFs) accessed from the NCBI database (GenBank accession number PRJNA241474). In South and Southeast Asia, chickens were domesticated from red jungle fowl (RJF) at least 4000–4500 years ago and have multiple origins within the geographic range of their wild ancestors [18]. The appearance and behavior of Tibetan chicken are very similar to the red jungle fowl [17].

### 2.2. Read Mapping, Genomic Variant Calling and Annotation

A quality check was performed for the low-quality paired sequences from the raw data and it was controlled using Trimmomatic software with the following parameters: (1) trimming of bases and N bases with sequence bipartite quality scores less than 20; (2) removal of sequence fragments with average base quality values below 20; and (3) removal of sequence fragments with trimmed lengths less than 30 bp. After the quality control, the clean reads were compared to the chicken reference genome (*Gallus gallus* 6.0) using BWA (version 0.7.15) with the command “mem -t 10 -k 32” [19], and then the BAM files were generated using SAMtools (version 0.1.19). Thereafter, the sequenced BAM files were de-duplicated by the sequence PCR using the ‘MarkDuplicates’ command in the Picard package (version 1.119). Further, gVCF calling was performed using the Genome Analysis Toolkit (GATK) (version v3.8) following the manufacturer’s instructions, then the best pipeline based on the HaplotypeCaller method was determined and population SNP calling was also conducted by the command ‘CombineGVCFs’ to merge all the gVCFs [20]. High confidence SNPs were obtained by applying the filtering command ‘VariantFiltration’, which excluded potential false positive variant calls, as follows: (a) quality by depth > 10.0; (b) mapping quality score > 40.0; (c) FS < 60.0; (d) MQRank-Sum > −12.5; (e) ReadPosRankSum > −8.0. In addition, the SNPs with an adjacency distance of ≤5 were removed. Then, the double-allelic variants were obtained with the following parameters using vcftools (version 0.1.15) [21]: --max-missing 0.9, --maf 0.05, --min-alleles 2, --max-alleles 2. High confidence variants were also retained from the 30 chickens. The SNPs were classified into different genomic regions (exonic regions, intronic regions, splice sites, upstream, downstream and intergenic regions) using ANNOVAR (version 20191024) [22].

### 2.3. Genome-Wide Selective Sweep Test

To ascertain genome-wide selective sweeps related to environmental adaptation, the genome-wide assignment of fixation index (FST) values and *pi* were calculated for the defined group pairs (vcftools v0.1.15). The average SNP FST values were plotted in 40 kb genomic bins with a 20 kb step. Then, the nucleotide diversity (*pi*) was estimated for the same bins. Furthermore, the FST values were Z-transformed as follows: Z (FST) = (FST − lFST)/rFST, in which lFST is the mean FST and rFST is the standard deviation of FST. *pi* was log2 transformed. Thereafter, the empirical percentiles of the Z (FST) and log2 (pi) were evaluated and arranged. The windows with the top 5% Z (FST) and log2 (pi) values were contemporaneously considered as the nominated outliers under strong selective sweeps.

### 2.4. Gene Ontology (GO) and KEGG Analysis

The candidate genes with positive signals obtained by selective clearance scans were uploaded into the Metascape [23] online tool for GO (Gene Ontology) functional analyses and KEGG (Kyoto Encyclopedia of Genes and Genomes) enrichment analyses. Only GO biological process (GO-BP), molecular function (GO-MF) and cellular component (GO-CC) enrichment analyses, as well as KEGG pathways, were considered significantly enriched (*p* < 0.05) to further explore the functional orientation of the specific candidate genes.

## 3. Results

### 3.1. Sample Information and Sequencing Results

In this study, a total of 327.49 Gb of raw data was sequenced. Firstly, the low-quality paired-ends were removed, and consequently high-quality paired-end reads were generated from the 30 samples with the quality of 96.73% and 91.93% with Q20 and Q30 bases, respectively.

The average sequencing depth of the 30 samples was ~7.95×, and the average comparison rate was ~98.65% (Table 1). A total of 9,490,690 SNPs were identified from the genomes of the 30 chickens. Furthermore, statistical annotation of the SNP (Table 2) showed that the ratio (Ts/Tv) of non-synonymous mutation to synonymous mutation in the population was 2.55, which reflected the quality of the SNP extraction, indicating that the identified SNPs detected in this study were accurate. In addition, we found that 112,547 SNPs were located upstream and 116,946 SNPs downstream, whereas 198,309 SNPs were located in the exon and most (about 68.62%) SNPs were synonymous mutations. In addition, 4,816,658 SNPs were located between genes and 5,977,177 SNPs (about 46.48%) were located in the intron region. Moreover, the annotation results showed that most SNPs were located in the non-coding regions, which is consistent with previous studies [24]. These distributions revealed that intergenic and intron regions in the genome play a key role in gene regulation and are responsible for the phenotypic diversity of chicken breeds.

### 3.2. Population Differentiation Based on Fst

**Figure 1:**
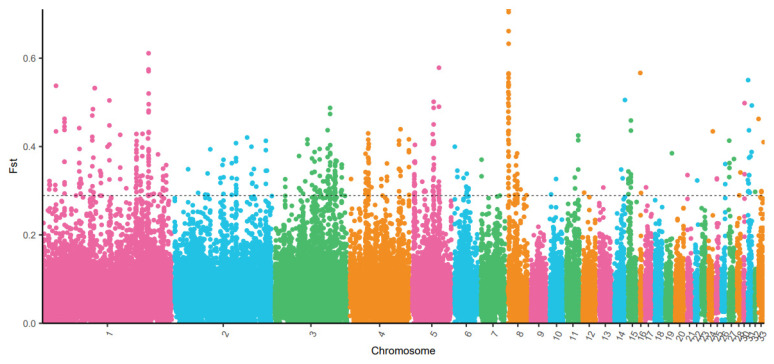
Distribution of Fst calculated using 40 kb windows with a 20 kb sliding window showing the genes identified and selected in the Tibetan black chicken (TBC) genome as compared with the red jungle fowl. Labeled genes have extreme Fst values (top 5% level) in the TBC selected genes.

The F-statistic reflects a change in the population structure, which is affected by different factors such as mutation, genetic drift, inbreeding, and selection or the Wahlund effect. According to the conditions of neutral evolution, the size of the F-statistic is mainly determined by influencing factors such as genetic drift and migration. If an allele in a population undergoes adaptive selection because of its high fitness in a specific habitat, then its frequency increases in the population. Subsequently, the level of population differentiation increases, which is reflected in the F-statistic (where 0 ≤ Fst ≤ 1). As the Fst was approximately 1, the differences between the subpopulation were greater. Figure 1 shows the distribution of the Fst in the genome of the Tibetan black chicken.

### 3.3. Sweeping Selection Based on Fst and Pi

**Figure 2:**
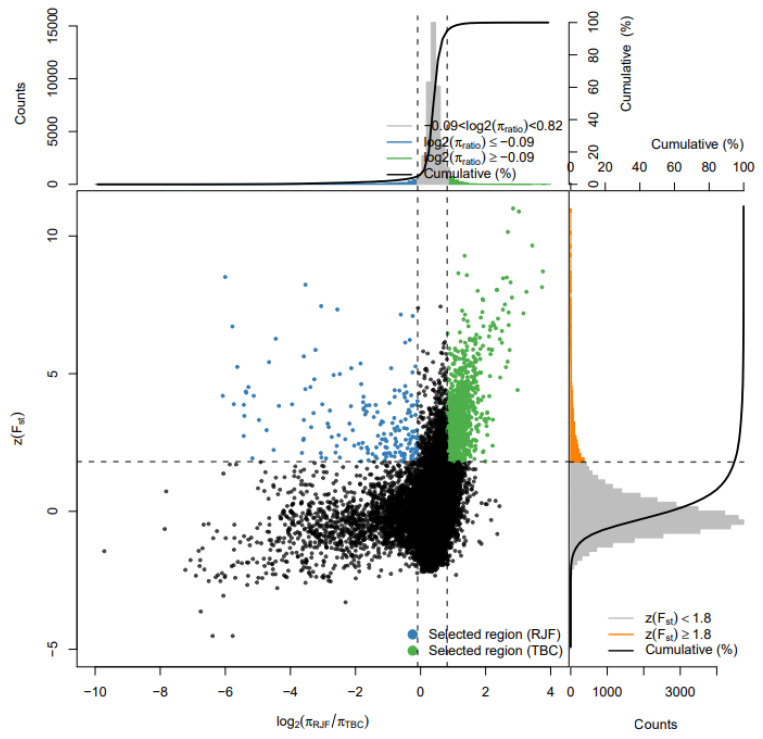
Fst and θπ combined in the screen selected regions. The abscissa is the ratio of θπ (red jungle chicken/Tibetan black chicken) and the ordinate is ZFst (Fst is standardized and converted to ZFst), which correspond to the frequency distribution diagram above and the frequency distribution diagram on the right, respectively. The dot diagram in the middle represents the corresponding ZFst and θπ ratios in different windows. The top blue and green areas are the top 5% area selected by θπ, and the orange area is the top 5% area selected by ZFst.

Fst and Pi (θπ or Pi or π) have been proven to be very effective in detecting and eliminating the areas under selection, especially when mining functional areas closely related to the living environment; a strong selection signal can often be obtained. Figure 2 shows the selection and elimination of Fst and elements. The results show that the joint screening for strong selection signals facilitates the screening of target genes.

### 3.4. GO and KEGG Enrichment Analysis

**Figure 3:**
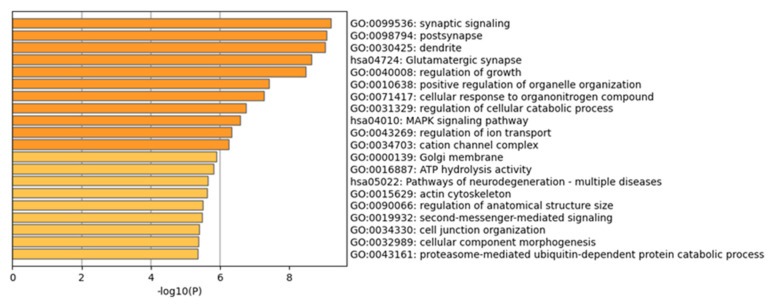
GO enrichment analysis of positive signals by subjected selection analysis method.

**Figure 4:**
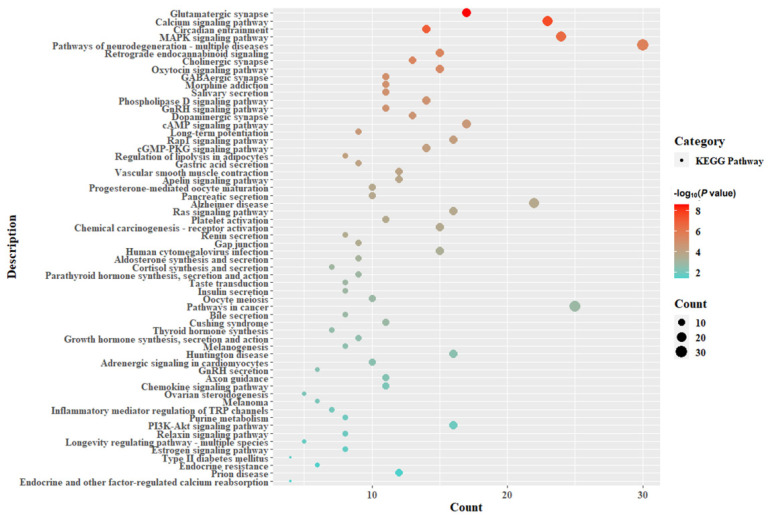
KEGG pathway analysis of positive signals.

The 732 candidate genes screened in the subject selection method were selected for KEGG pathway (Figure 3 and Supplementary Table S1) and GO enrichment analyses (Figure 4). The results obtained from the KEGG pathway analysis showed significant biological pathways such as calcium signaling pathways, circadian regulation, the MAPK signaling pathway, the GnRH signaling pathway, neurodegenerative pathway multiple diseases, the oxytocin signaling pathway, salivary secretion, the phospholipase D signaling pathway, gastric acid secretion, vascular smooth muscle contraction, progesterone-mediated oocyte maturation, oocyte meiosis, melanogenesis, thyroid hormone synthesis and growth hormone synthesis. However, the GO enrichment analysis was mainly concentrated in synaptic signaling, post synapse, dendrite, glutamatergic synapse, regulation of growth, positive regulation of organelle organization, cellular response to organonitrogen compound, regulation of cellular catabolic process, the MAPK signaling pathway, regulation of ion transport, cation channel complexes, Golgi membranes, ATP hydrolysis activity, pathways of neurodegeneration—multiple diseases, the actin cytoskeleton, regulation of anatomical structure size, second-messenger-mediated signaling, cell junction organization, cellular component morphogenesis and the proteasome-mediated ubiquitin-dependent protein catabolic process.

## 4. Discussion

### 4.1. Functional Annotation of TBCs

The footprints of the genomic selection were determined by comparing the genome variation of TBC with RJF, where genes characterized with strong selective sweep signals were screened (Supplementary Table S2). A total of 732 genes were enriched in 210 GO terms in the TBCs, and the top 20 GO terms are listed in Figure 4. Among the top 20 GO terms, terms such as GO: 0048589 and GO: 0060560 were associated with growth, whereas GO:0048286 (lung alveolus development) was associated with alveolar development, which may provide a better mechanism to facilitate oxygen transport and utilization, thereby improving adaptation to specific environments of TBCs. This finding indicates that TBCs could adapt and survive in high-altitude and high-humidity environments. The TBCs are raised in a low-temperature environment with an annual average temperature of 5.5 °C [1]. The phospholipase D signaling pathway (*ADCY1*, *ADCY6*, *ADCY8*, *GRM5*, *GRM7*, *PLA2G4A*, *PLCB4*, *PLD1*, *DGKI*, *AKT3*, *MRAS*, *PLCB1*, *AGPAT4* and *PDGFD*) was detected. The top 10 genes that were selected for censoring based on Fst and *Pi* scanning were *ENSGALG00000046923*, *AMY2A*, *TSHR*, *GTF2A1*, *GPR156*, *GSK3B*, *ENSGALG00000052399*, *ENSGALG00000052957*, *ENSGALG00000048440* and *STXBP6*. TSHR is responsible for regulating thyroid function. Studies have reported that *TSHR* is associated with an increased egg production in domestic chickens [25] and pleiotropic phenotypes, including behavioral and environmental adaptive traits [26]. Studies have shown that similar selection pressures influenced domestic chickens in Ethiopia, Saudi Arabia and Sri Lanka [27].

Growth factor-stimulated phospholipase D (*PLD*) is known to catalyze the hydrolysis of phosphatidylcholine (PC) to generate phosphatidic acid (PA), which acts as a second messenger during cell proliferation and survival. *PLD* is also believed to play an important role in tumorigenesis [28], as well as in regulating cell proliferation and tumorigenesis [29]. Therefore, due to the specific growth environment of the TBC, the phospholipase D signaling pathway could be responsible for disease resistance in these chickens. *BDNF* and *APBA2* are involved in the regulation of neurological and behavioral traits such as aggression. The *KIF18A* gene functions in mitotic expansion and sperm cell differentiation during testicular maturation [30]. In the present study, the protein interaction network analysis showed that the *KIF18A* gene was significantly enriched in oocyte meiosis. Previous studies have shown that the disruption of this gene causes sterility in mice [31]. In addition, studies have reported that the *GNRH-1* and *KIF18A* genes play key roles in increasing fertility, as well as in reducing the time to sexual maturity in domestic chickens [32]. Furthermore, other studies have revealed that *VCL* is associated with muscle development and cardiovascular activity [33].

### 4.2. Potential Disease Resistance in TBCs

The muscle development and growth of TBCs and RJFs are significantly different due to artificial selection. Candidate regions for traits related to growth contain several genes, including sphingomyelin phosphodiesterase 3 (*SMPD3*), which regulates cell growth, differentiation and apoptosis, as well as promoting postnatal growth and development [34]. In addition, neuroectodermal growth factor-like protein 1 (*NELL1*) encodes a cytoplasmic protein containing an epidermal growth factor-like repeat sequence and also targets the osteochondral spectrum [35]. In contrast to RJF, the *SMPD3* and *NELL1* genes may be associated with growth performance in poultry and weight gain in TBCs. Furthermore, *AKT3* is known to play an active role in the survival of human malignant glioma cells [36]. The activation of the *ADRB2/PKA* signaling pathway promotes lipid synthesis in human lid gland epithelial cells and also provides a potential mechanism and therapeutic target for dry eye disease [37]. Previous reports have indicated that cell-death-inducing DNA fragmentation factor-α-like effector C (*CIDEC*) is positively relates to insulin sensitivity, whereas the expression of endothelial-specific *CIDEC* improves vascular and metabolic dysfunction induced by a high-fat diet [38]. Bi-allelic *SLC4A2* loss-of-function mutations in mice and cattle lead to osteopetrosis with osteoclast deficiency [39]. These genes may be responsible for disease resistance traits in TBCs. In the present study, the CD86 antigen (*CD86*) was identified as a gene associated with immune response. It is a well-known immune-related gene associated with cytotoxic T-lymphocyte antigen 4 (CTLA-4), which negatively regulates the T-cell immune response [40]. These findings support the trade-off between selecting for growth and immunity during chicken domestication [41]. Genes such as *AMY1A*, *AMY1B*, *AMY1C*, *AMY2A* and *AMY2B* were coded with the starch digestion enzyme called amylase. The copy number variation (CNV) of *AMY1* has been implicated in human dietary adaptation and in population association with obesity [42]. Previous studies have demonstrated new functional annotations enhance biological interpretation of complex traits and human diseases [43]. Previous studies showed that whole-genome resequencing patterns in human amylase variations imply a potential role of *AMY2* CNV in functional associations [42]. However, further studies are needed to verify this. It is important to understand the mechanisms underlying the traits of economic importance and adaptability through functional annotation of the genome of animals [43]. It was reported that the pig genome revealed the genetic relationship among variants of domestic ducks with their potential wild ancestors in eastern China, as well as revealing how their genomes were shaped by different natural and artificial selective pressures. Several candidate genes and important pathways related to artificial selection were also identified which were functionally related to lipid metabolism, muscle functioning, the cell cycle, type 2 diabetes, cellular adhesion and liver cell proliferation in domestic ducks [44]. In other studies, genome-wide detection of selective signatures in chickens showed a panel of genes, including *AASDHPPT*, *GDPD5*, *PAR3*, *SOX6*, *GPC1* and a signal pathway of AKT1, which were associated with the immune function, sensory organ development and neurogenesis and may have experienced positive selection in chickens [45]. This therefore draws a comparatively integrated genome-wide map of the selection signature in the chicken genome [45]. In this present study, several significant biological pathways such as circadian regulation, neurodegenerative pathway multiple diseases, the oxytocin signaling pathway, progesterone-mediated oocyte maturation, oocyte meiosis, melanogenesis, thyroid hormone synthesis and growth hormone synthesis were identified.

### 4.3. High-Altitude Adaptation in TBCs

In this present study, four genes (*TRIT1*, *HPCAL4*, *NT5C1A* and *HEYL*) were discovered under selection in the Tibetan black chickens on chromosome 23. These genes may be related to the local adaptative characteristic of Tibetan black chickens. *TRIT1* is a tumor suppressor, which inhibits cell growth [46] and could be responsible for the dwarf phenotype of Tibetan chickens. In addition, the protein encoded by the *HPCAL4* gene is a Ca^2+^-binding protein which is similar to the human hippocalcin protein and hippocalcin-like 1 protein. Studies have indicated that human cytosolic 5′-nucleotidase is highly expressed in both the skeletal and heart muscles [47] and it was reported to play key role in the metabolism of purine, pyrimidine, nicotinate and nicotinamide. In addition, cytosolic 5′-nucleotidase 1A (*NT5C1A*) was found to regulate body fluid levels, and it is a transcript that is differentially expressed in high-altitude areas to promote efficient oxygen usage [48]. Furthermore, *HEYL* regulates specific sets of cardiac genes via regulating the role of GATA protein and other cardiac transcriptional activators in a signal-dependent manner [49]. Tibetan black chickens living in a high-altitude area are characterized with increased hemoglobin concentrations, blood oxygen affinities and red blood cell counts, as well as a decreased mean corpuscular volume, to overcome harsh conditions in their environment [50]. This indicates that *NT5C1A* and *HEYL* may be involved in the adaptation and survival of Tibetan black chickens in high-altitude, oxygen-deficient areas. Candidate genes located in the calcium-signaling pathway may be associated with the hypoxia adaptation in Tibetan black chickens [15]. In this present study, these genes were mainly associated with energy metabolism, immune response, growth regulation and skeletal muscle development. These selection signals found are involved in the immune response, high-altitude adaption of oxygen delivery and genetic features caused by selective breeding, which were strongly correlated with some important economical traits that are of particular value to the poultry industry, including egg and meat yields. These findings are beneficial as they provide useful genetic information on the Tibetan black chicken that is conducive to the screening and utilization of genes related to important economic traits, as well as providing genetic information for the use in breeding, and they could shed light on the evolutionary history of the Tibetan black chicken. In the Tibetan black chicken, several biological processes are involved in GTPase regulation activities, which shows that energy metabolism is important to maintain the body temperature of the chickens. In addition, *NT5C1A* regulates the adenosine levels in the heart during hypoxia and ischemia, especially when blood circulation throughout the body is inadequate [51].

Other studies have also shown that *SMPD3* phingomyelin phosphodiesterase regulates cell growth, differentiation and apoptosis and contributes to postnatal growth and development [52]. The inactivation of *SMPD3* causes skeletal malformations, whereas *SMPD3* deficiency can result in juvenile dwarfism in neutral sphingomyelinase (*SMPD3*) mice, suggesting that *SMPD3* is a polygenetic determinant of body height [53]. *NDUFS2*, a Core Subunit of Mitochondrial Complex I, is essential for acute oxygen sensing and hypoxic pulmonary vasoconstriction [54]. This shows that these specific genes provide a better mechanism to facilitate oxygen transport and utilization in TBC, thereby improving adaptation to specific environments.

### 4.4. Egg Production

The TBC is a dual-purpose breed raised for both meat and eggs. The highly differentiated peaks in the selected regions contained *KIF18A* and *GTF2A1* genes, which are required for mitotic progression during germline development [55]. A previous study indicated that the *KIF18A* gene improves egg production in Tianfu black-boned chickens and Peng county yellow chickens, whereas *GTF2A1* is considered as a candidate gene associated with egg production [56].

### 4.5. Aggressiveness

We observed in this study that genes such as *BDNF* and *APBA2* were highly differentiated at peaks in the selected regions. The results showed that *BDNF* and *APBA2* are involved in the regulation of neurological and behavioral traits, such as aggression; however, *BDNF* affects prenatal priming on developing species-specific behaviors [57]. Studies have shown that fibroblast growth factor 2 (*FGF2*), glial cell-line-derived neurotrophic factor (*GDNF)* and brain-derived neurotrophic factor (*BDNF*) play roles in the survival of midbrain dopaminergic neurons of chickens [58]. A mutation in the human *BDNF* gene has been reported to be correlated with aggressive behavior. Reports have indicated that a loss of function of *BDNF* paves the way for studying aggression in animals. In Xishuangbanna fighting cocks, *BDNF* was screened to reveal chicken aggressiveness [59]. This shows that TBCs have some degree of aggressiveness. Aggression has been linked to wild relatives, partly due to predator avoidance and sexual selection behaviors, which can be seen as positive selection in most domestic chicken breeds [27].

### 4.6. Potentially Selected Genes Related to Vision, Hearing and Circadian Rhythms

Vision in animals is a key ability that influences their survivability. It affects behavioral traits such as defense, foraging, predator avoidance and mating [60]. Positive selection promotes the evolution of vision during chicken domestication. Positive selection can drive the regulation of gene expression, which attenuates the vision of chicken [61]. *IFT140* (intraflagellar transport 140) encodes a sub-unit of the intraflagellar transport complex A, and it can play an important role in optin transport, which is essential for light detection [62]. Intraflagellar transport 57 (*IFT57*) has also been reported to be involved in the regulation of photoreceptors [63]. *SPARC* (secreted protein acidic and rich in cysteine)-related modular calcium binding 1 (*SMOC1*) is essential for ocular and limb development in both humans and mice [64]. Hearing loss can be caused by mutations of certain G protein-coupled receptors (GPCRs), such as *GPR156* [65]. In addition, circadian regulation genes (*ADCY1*, *GNG7* and *PER3*) were significantly enriched. Adenylate cyclase 1 (*ADCY1*) mutations cause recessive hearing impairment in humans and defects in hair cell function and hearing in zebrafish [66]. Preeclampsia (PE) is a hypertensive disorder of uncertain etiology that is the leading cause of maternal and fetal morbidity or mortality. Upregulation of *GNG7* exerts an inhibitory effect in PE [67]. *Per3* knockout mice have phenotypes related to activity, sleep homeostasis, anhedonia, metabolism and behavioral responses to light [68]. The regulation of circadian rhythms may be related to the long average annual daylight hours in the living environment; however, the exact mechanism requires further study.

## 5. Conclusions

In this study, the genetic multiplicity of Tibetan black chicken was analyzed using whole-genome resequencing. We found the loci of genetic diversity and the selective signals in the TBC. A comparison of the genome sequences of the Tibetan black chicken and the red jungle fowl showed the distinct scenarios of species evolution under natural selection; hence, survival is promoted under high altitude and humidity conditions and with artificial selection for egg production. In this present study, several candidate genes were found to be associated with aggressiveness and positive selection for vision in Tibetan black chickens. In addition, we found that the CD86 antigen (*CD86*) gene was associated with the immune response in chickens. It was also revealed that genes such as *TRIT1*, *HPCAL4*, *NT5C1A* and *HEYL* were discovered under selection in the Tibetan black chickens on chromosome 23. These genes may be related to the local adaptive characteristics of Tibetan black chickens, for instance, *NT5C1A* and *HEYL* may be involved in the high-altitude adaption of oxygen delivery in Tibetan black chickens. This study provides relevant information on the genomic data for the evolution and breeding of Tibetan black chickens. In addition, the results provide a theoretical basis for the protection, popularization and utilization of Tibetan black chickens. This study provides basic genetic information for the evolution and breeding of Tibetan black chickens, thereby determining high-quality traits in this chicken breed.

## Figures and Tables

**Table 1:** Statistics of whole-genome data of the Tibetan black chicken.

Sample Name	Clean Reads	Coverage Depth (X)	Raw Base (G)	Clean Base (G)	Effective Rate (%)	Q20 (%)	Q30(%)	GC Content (%)
1	70346492	8.59	10.57	10.55	99.83	95.95	89.99	43.21
2	67577374	7.33	10.16	10.14	99.74	97.47	93.6	42.88
3	77612348	9.67	11.66	11.64	99.84	96.13	89.64	42.36
4	65717850	6.81	9.88	9.86	99.76	97.53	93.59	42.97
5	69322110	7.47	10.42	10.40	99.76	97.32	93.16	42.37
6	67171880	7.1	10.10	10.08	99.7	97.08	92.73	43.00
7	73091286	7.36	10.99	10.96	99.72	97.33	93.22	42.52
8	70688764	7.57	10.64	10.60	99.67	97.22	93.09	42.09
9	66844024	7.32	10.06	10.03	99.7	96.69	91.73	42.27
10	94968550	10.64	14.27	14.25	99.84	96.68	91.56	42.56
11	69673108	7.55	10.48	10.45	99.74	97.14	93.16	42.95
12	75912388	9.31	11.41	11.39	99.84	95.82	89.75	42.7
13	77491138	9.36	11.64	11.62	99.84	95.78	89.57	42.77
14	71980470	8.86	10.81	10.80	99.84	96.34	90.7	42.55
15	76923340	8.22	11.58	11.54	99.67	97.00	92.81	43.37
16	71735168	7.71	10.79	10.76	99.74	97.11	92.79	43.77
17	97405862	11.71	14.64	14.61	99.83	96.02	90.19	42.49
18	70090036	7.47	10.55	10.51	99.59	96.48	91.77	43.13
19	72877790	7.7	10.97	10.93	99.65	96.67	91.97	42.9
20	70938848	8.06	10.67	10.64	99.69	96.94	92.42	42.81
21	70079014	7.53	10.55	10.51	99.6	96.64	91.98	42.7
22	64508976	6.96	9.70	9.68	99.7	97.25	93.19	43.27
23	68653894	7.35	10.32	10.30	99.73	97.44	93.53	43.22
24	69469802	7.54	10.45	10.42	99.69	97.06	92.9	43.64
25	68040032	7.23	10.23	10.21	99.71	96.8	92.13	42.65
26	67368530	7.24	10.13	10.11	99.73	96.78	92.06	42.53
27	84735694	10.4	12.73	12.71	99.84	95.58	89.35	42.33
28	63404212	3.04	9.56	9.51	99.51	95.61	90.52	48.93
29	71416314	7.56	10.74	10.71	99.73	97.22	92.92	42.52
30	71800010	7.85	10.79	10.77	99.74	96.81	91.97	42.65

**Table 2:** Distribution of SNPs in the gene functional regions.

Total SNP		9,490,690	100%
Upstream		112,547	1.19%
Exonic	Nonsynonymous	91,261	2.09%
	Stopgain	424	
	Stoploss	68	
	Synonymous	106,556	
Intronic		4,427,978	46.66%
Upstream/Downstream		7921	0.08%
Downstream		116,946	1.23%
Intergenic		4,565,787	48.11%
Transitions		6,814,921	
Transversions		2,675,769	
Transitions/Transversions		2.55	

Upstream: variants that overlap the 1 kb region upstream of the gene start site. Stop gain: nonsynonymous SNPs lead to the generation of stop codons at the mutated site. Stop loss: a non-synonymous SNP results in the disappearance of the stop codon at the mutated site. Downstream: a variant overlapped at the 1-kb region downstream of the gene end position. Upstream/Downstream: a variant was located in the downstream and upstream regions (possibly two different genes).

## Data Availability

The accession number for the genome re-sequencing data reported in this paper is NCBI SRA: PRJNA967028. Other data that supported the results presented in this study can be obtained from the corresponding authors.

## Supplementary Materials

The following supporting information can be downloaded at: https://www.mdpi.com/article/10.3390/genes14091672/s1, Table S1: Summary of the selected genes across the Fst and pi values in the TBCs; Table S2: The “Annotation” of gene identifiers, annotations, binary membership search and enrichment results (1/0), and the “Enrichment” of all enriched terms and their cluster group IDs.
